# Editorial: Coronary physiology in the spotlight: advancing diagnosis and treatment in CAD and microvascular disease

**DOI:** 10.3389/fcvm.2026.1794772

**Published:** 2026-02-13

**Authors:** Nikolaos Stalikas, Efstratios Karagiannidis, Frederic Bouisset

**Affiliations:** 1 Cardiovascular Center Aalst, AZORG Hospital, Aalst, Belgium; 2 Department of Emergency Medicine, Aristotle University of Thessaloniki, AHEPA University Hospital, Thessaloniki, Greece; 3 Department of Cardiology, Toulouse University Hospital, Toulouse, France

**Keywords:** CMD, coronary physiology, CT-FFR, FFR, inflammation

Coronary artery disease (CAD) remains a leading cause of morbidity and mortality worldwide, despite remarkable advances in imaging, pharmacotherapy, and revascularization strategies (1). For decades, clinical decision-making has largely relied on anatomical assessment of epicardial stenoses. However, it has become increasingly evident that coronary anatomy alone incompletely captures ischemic burden, biological risk, and long-term prognosis (2). This Research Topic was conceived to place coronary physiology at the center of contemporary CAD management, highlighting how functional assessment, plaque biology, and coronary microvascular disease (CMD) together refine diagnosis, guide therapy, and improve risk stratification.

## Beyond stenosis: integrating physiology and plaque biology

Among the most transformative contributions in this Topic is the growing role of non-invasive coronary physiology derived from *coronary computed tomography angiography (CTA)*. The prospective cohort study combining AI-derived CT fractional flow reserve (CT-FFR) with high-risk plaque (HRP) characteristics exemplifies this evolution. By demonstrating that the integration of functional ischemia and HRP features markedly improves prediction of major adverse cardiovascular events (MACE) compared with either metric alone, this work reinforces a crucial concept: ischemia and vulnerability are complementary rather than competing pathways of risk.

This integrated approach moves CTA from a purely anatomical test to a comprehensive platform for precision risk assessment at the time of diagnosis. Beyond anatomical and non-invasive physiological assessment, emerging evidence suggests that CTA can also inform interventionalists about key procedural characteristics. CTA enables comprehensive procedural planning by integrating lesion-specific physiological significance, myocardial mass at risk, plaque composition and distribution, three-dimensional plaque mapping, optimal projection selection, catheter choice, and CT-derived 3D guidance (3, 4). Importantly, the ability to derive anatomical, physiological, and procedural information from a single examination substantially enhances feasibility, supporting a future paradigm in which invasive angiography may be reserved for patients in whom anatomy, physiology, and biology converge toward percutaneous coronary intervention (PCI).

## Microvascular dysfunction: the missing link after revascularization

While epicardial disease remains the most visible manifestation of CAD, coronary microvascular dysfunction has emerged as a key determinant of persistent symptoms and adverse outcomes. *The systematic review and meta-analysis included in this Topic* provides compelling evidence that CMD affects approximately 40% of post-PCI target vessels and is independently associated with a substantially increased risk of MACE.

This finding has major clinical implications. It explains, at least in part, why technically successful PCI does not always translate into symptomatic relief or prognostic benefit. By consolidating data across diverse populations and physiological assessment methods, this meta-analysis establishes CMD as a frequent and prognostically meaningful entity that warrants systematic evaluation.

Mechanistic insights are further expanded by original work on *coronary wave intensity analysis*, which explores microvascular-originated backward waves and their modulation under varying hemodynamic conditions. These data enhance our understanding of myocardial-coronary coupling and support the clinical feasibility of advanced physiological indices capable of interrogating microvascular status.

Equally important, the clinical translation of advanced physiological indices requires robust validation of measurement agreement and reproducibility. Within this Research Topic, a *dedicated methodological study employing Bland–Altman analysis* addresses this critical aspect by evaluating the agreement between physiological measurements across modalities and analytical approaches. By demonstrating acceptable limits of agreement and highlighting sources of variability, this work reinforces confidence in the interpretability and interchangeability of contemporary physiological tools.

## Physiology-guided therapy and procedural optimization

Several contributions in this Topic emphasize the role of physiology not only in diagnosis but also in guiding interventional strategy. *The study evaluating quantitative flow ratio (QFR) in drug-coated balloon (DCB) therapy* illustrates how functional assessment after lesion preparation can predict mid-term vessel physiology and identify patients at risk for residual functional stenosis. Such findings reinforce the concept that physiological optimization should extend beyond stent deployment and may be particularly relevant in “leave-nothing behind” revascularization strategies.

## Inflammation, biomarkers, and functional risk

Coronary physiology does not operate in isolation from systemic biology. *The investigation of the mean platelet volume-to-monocyte ratio (MMR)* highlights the complex interplay between inflammation, thrombosis, and coronary outcomes. The observed nonlinear association between MMR and long-term prognosis underscores the limitations of single-threshold biomarkers and aligns with emerging evidence that inflammatory activity modulates both plaque behavior and microvascular function.

Beyond inflammatory indices, dysregulation of endogenous anticoagulant pathways may further modulate ischemic risk and coronary physiology. In this Research Topic, *a dedicated study examining plasma antithrombin levels* highlights the prognostic relevance of hemostatic balance in patients with coronary artery disease.

## Innovative tools for early detection and risk stratification

This Research Topic also explores novel, less conventional approaches to CAD detection and prediction. *A meta-analysis evaluating a phonocardiogram-based CAD score* demonstrates the potential of acoustic signal analysis as a low-cost, non-invasive rule-out tool in patients with suspected CAD. While not a substitute for imaging or physiology, such approaches may complement existing pathways by improving pre-test probability assessment and reducing unnecessary testing.

Similarly, *the development of a LASSO-based predictive nomogram for obstructive CAD* in patients with repeatedly zero calcium scores challenges the notion of “low-risk” anatomy. By identifying clinical and metabolic predictors of disease progression despite favorable imaging findings, this work highlights the limitations of static anatomical risk models and reinforces the need for dynamic, personalized assessment.

## Toward precision coronary medicine

Collectively, the studies in this Research Topic illustrate a unifying message: coronary physiology is no longer an adjunct but a foundation of modern CAD care. From CT-derived functional imaging and plaque characterization, to invasive and angiography-based physiological indices, to microvascular assessment and systemic biomarkers, the field is moving toward an integrated, patient-centered model of coronary disease. By bringing these diverse yet complementary perspectives together, this Research Topic aims to advance the ongoing transition from anatomy-driven intervention to precision coronary medicine.
